# First results of undersea muography with the Tokyo-Bay Seafloor Hyper-Kilometric Submarine Deep Detector

**DOI:** 10.1038/s41598-021-98559-8

**Published:** 2021-09-30

**Authors:** Hiroyuki K. M. Tanaka, Masaatsu Aichi, Cristiano Bozza, Rosa Coniglione, Jon Gluyas, Naoto Hayashi, Marko Holma, Osamu Kamoshida, Yasuhiro Kato, Tadahiro Kin, Pasi Kuusiniemi, Giovanni Leone, Domenico Lo Presti, Jun Matsushima, Hideaki Miyamoto, Hirohisa Mori, Yukihiro Nomura, László Oláh, Sara Steigerwald, Kenji Shimazoe, Kenji Sumiya, Hiroyuki Takahashi, Lee F. Thompson, Yusuke Yokota, Sean Paling, Dezső Varga

**Affiliations:** 1 University of Tokyo, Tokyo, Japan; 2 The University of Salerno, Salerno, Italy; 3 Istituto Nazionale di Fisica Nucleare-Laboratori Nazionali del Sud, Catania, Italy; 4 Durham University, Durham, UK; 5 Kerttu Saalasti Institute, University of Oulu, Oulu, Finland; 6 Muon Solutions Oy Ltd, Saarenkylä, Finland; 7 Arctic Planetary Science Institute, Rovaniemi, Finland; 8 NEC Corporation, Tokyo, Japan; 9 Kyushu University, Fukuoka, Japan; 10 The University of Atacama, Copiapó, Chile; 11 The University of Catania, Catania, Italy; 12 Istituto Nazionale di Fisica Nucleare, Catania, Italy; 13 Kansai University, Osaka, Japan; 14 The University of Sheffield, Sheffield, UK; 15 Geoptic Ltd, London, UK; 16 Boulby Underground Laboratory, Loftus, UK; 17 Wigner Research Centre for Physics, Budapest, Hungary; 18 International Virtual Muography Institute (VMI), Global, Tokyo, Japan

**Keywords:** Physical oceanography, Ocean sciences, Physics, Particle physics, Experimental particle physics, Solid Earth sciences, Geophysics

## Abstract

Tidal measurements are of great significance since they may provide us with essential data to apply towards protection of coastal communities and sea traffic. Currently, tide gauge stations and laser altimetry are commonly used for these measurements. On the other hand, muography sensors can be located underneath the seafloor inside an undersea tunnel where electric and telecommunication infrastructures are more readily available. In this work, the world’s first under-seafloor particle detector array called the Tokyo-bay Seafloor Hyper-Kilometric Submarine Deep Detector (TS-HKMSDD) was deployed underneath the Tokyo-Bay seafloor for conducting submarine muography. The resultant 80-day consecutive time-sequential muographic data were converted to the tidal levels based on the parameters determined from the first-day astronomical tide height (ATH) data. The standard deviation between ATH and muographic results for the rest of a 79-day measurement period was 12.85 cm. We anticipate that if the length of the TS-HKMSDD is extended from 100 m to a full-scale as large as 9.6 km to provide continuous tidal information along the tunnel, this muography application will become an established standard, demonstrating its effectiveness as practical tide monitor for this heavy traffic waterway in Tokyo and in other important sea traffic areas worldwide.

## Introduction

Sea levels have been continuously measured and modeled extensively in the last decades in order to evaluate the global and local climate change and ocean variability. In particular, for coastal communities, reliable sea-level information is essential since coastal flooding is increasingly occurring in many areas^1,2^. The heights of coastal barriers or breakwaters are typically designed by considering the superimposed effects of tides, surges, waves, and relative sea-level rise^3^. However, even within the Tokyo metropolitan bay area, one of the most populated areas in the world, TGSs are still sparsely distributed; along the 770 km of coast making up the Tokyo bay of Japan, there are only four TGSs with long-term data taking capabilities.

Remote sensing techniques provide a more flexible solution for analyzing sea level changes in comparison to TGSs. The main remote sensing technique used is Radar altimetry. This technique that combines the information about the predicted height of the satellite with geophysical corrections provides the reflecting point on the sea surface. Such information has been used for monitoring sea level rise and geostrophic currents in the ocean^4^. Global-navigation-satellite-system (GNSS) buoys are a new type of a tide gauge that has been developed with GNSS satellite positioning technology, allowing us to distribute the tide gauges in the offshore region, and it can measure tidal level with high precision and relatively fast time resolutions (30 s–1 day)^5^. However, generally, buoys equipped with GNSS are used to derive the mean sea surface that is averaged over time. Also, GNSS can be mounted in a buoy to measure the sea surface height (SSH) referred to ellipsoid. However, the SSH contains the geoid. Moreover, installing a large array of GNSS buoys in the high traffic coastal areas is not practical due to the obstruction it would create to sea traffic and the high cost of deployment.

Ocean bottom pressure gauging and ultrasonic gauges are also alternative methods that have been developed for detecting the sea level changes from the seafloor^6^. However, the pressure gauging technique has an intrinsic drift error, and moreover, the sensors have to be directly located on the seafloor, and thus it is challenging to prepare the electric and telecommunication network that are required for real-time monitoring. Like pressure gauges, ultrasonic gauges do not have to float on the sea surface, and there are no intrinsic drift errors either. This technique measures the propagation time of the ultrasonic signals between the sea surface and the sensor located at the seafloor. However, in particular, at shallow depths of seawater near coastal areas, sound propagation is strongly affected by solar radiation, seasonal cycles, mixing of the water due to sea currents, and the presence of rivers or waste waters^7^. On the contrary, the muon propagation properties are much less sensitive to the oceanic environment, e.g., variations in salinity, temperature, internal density waves in the upper-ocean, or motor sound in comparison to techniques that measure acoustic signals^8^. More importantly, since muons propagate through solid and liquid materials in a similar manner, the sensors can be located underneath the seafloor; for example, muon detectors can be placed inside an undersea tunnel where electric and telecommunication infrastructures are not only convenient and well-arranged but often readily available and an important part of the original tunnel design.

Cosmic-ray muons are relativistic particles with a rest mass of 105 MeV. They travel almost at the speed of light through any kind of material and their speed is not affected by the media condition they travel as long as their energies are within the relativistic region. They are produced in the Earth's atmosphere via the collision between primary cosmic rays, which are mainly galactic cosmic rays (GCRs), and atmospheric nuclei, such as nitrogen and oxygen. These particles are ubiquitous and thus, available everywhere on the Earth. GCRs arrive at the Earth isotropically, because before arriving at the Earth, GCRs are deflected multiple times during their propagation in the galaxy, and lose their initial directional information. However, due to different atmospheric thicknesses and density gradients for different GCR's arrival angles, the muon energy spectrum varies for different zenith angles. As a consequence, the vertical muon flux is higher than the horizontal flux, but the average energy of vertical muons is lower than the horizontal ones. Muons are called heavy electrons. Due to the mass of a muon, they experience much less radiative energy loss processes (Bremsstrahlung, direct pair production, and photonuclear interaction) than electrons, and consequently are much more penetrative than electrons. In conjunction with this penetrative nature and this universality, muography has been widely applied to visualizing the internal structure of gigantic objects such as volcanoes, highway and railway tunnels, natural caves, and cultural heritage in Africa^9,10^, the Americas^11–13^, Asia^14–22^, and Europe^23–29^.

Undersea particle detectors have been deployed on the seafloor at large depths: in the past DUMAND^30^ and presently ANTARES^31^ and the detector KM3NeT^32,33^. However, it is difficult to utilize them for the purpose of muography because they are generally located at deep sea. Firstly, signal to noise ratio (tidal variation/total thickness of water) is very small. Secondly, at depths of 2–3 km underwater, the number of muons in a 5 min time slice is ~ 15^34^. This would yield a 25% relative uncertainty that is equivalent to an uncertainty in determining the sea level of 50–80 m.

**Figure 1:**
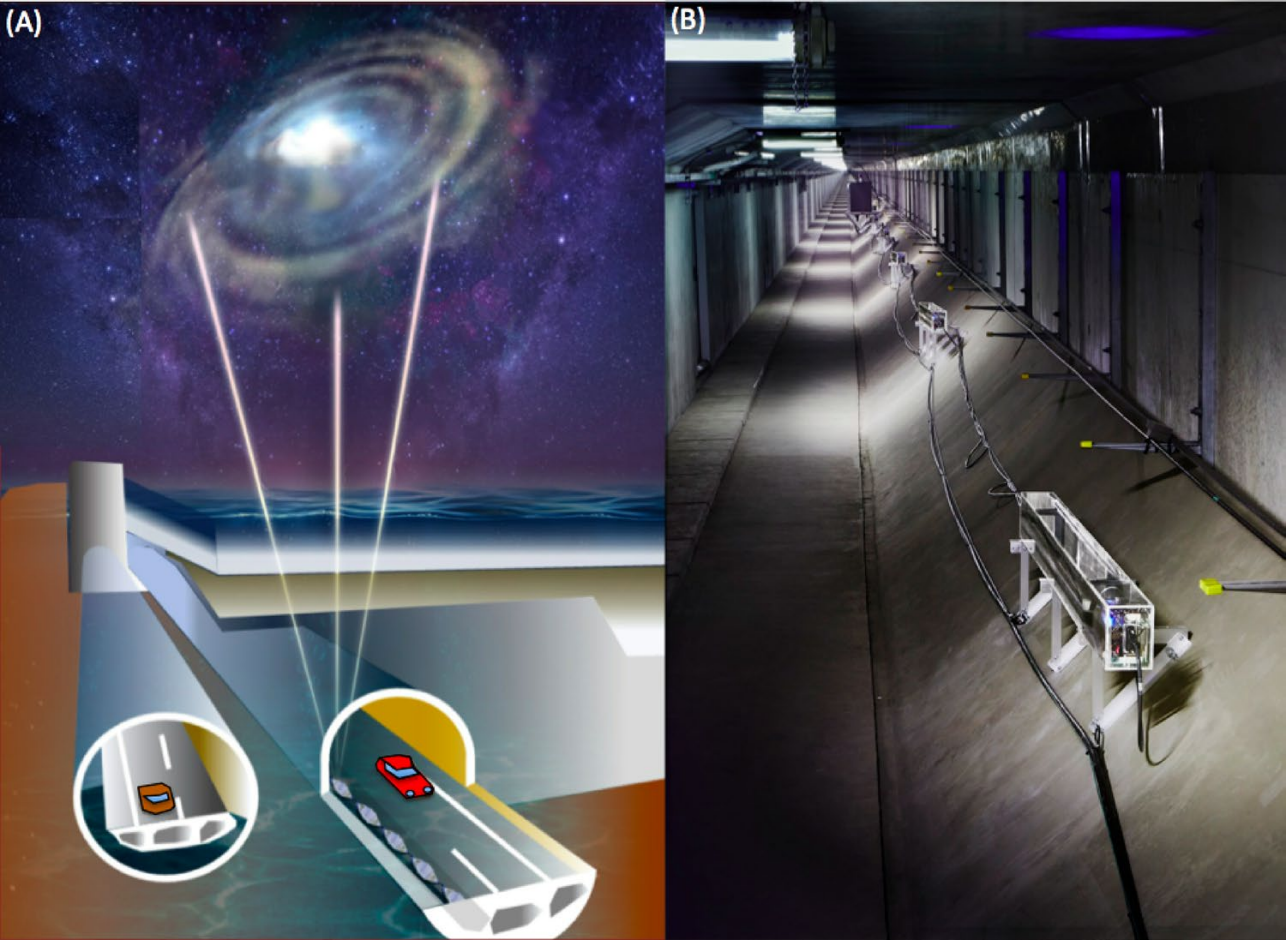
Conceptual view of the Tokyo-bay Seafloor Hyper-Kilometric Submarine Deep Detector (TS-HKMSDD) deployed underneath the Tokyo bay seafloor (**A**) and the photograph at the actual site (**B**). HKMT drew this image and holds the copyright. HKMT holds the copyright of the photograph.

The world’s first under-seafloor detector array for conducting submarine muography called the Tokyo-bay Seafloor Hyper-Kilometric Submarine Deep Detector (TS-HKMSDD) was deployed underneath the Tokyo bay seafloor on March 5, 2021 (Fig. 1). The ten installed muon sensor modules (MSMs) were anchor-bolted inside the undersea tunnel called the Tokyo-bay Aqua-tunnel; this tunnel provides an ideal environment for submarine/under-seafloor muography and overcomes the aforementioned issues. The detector collects near vertical muons after they have passed through the seawater and upper layer of the seabed of Tokyo-bay existing directly above the tunnel. The time-sequential muographic data were collected and converted to the tide levels above the detector. Then these results were compared with the astronomical tide height (ATH). The potential and prospects of this first combination of muography in the undersea tunnel environment is discussed in the following sections.

## Results

**Figure 2:**
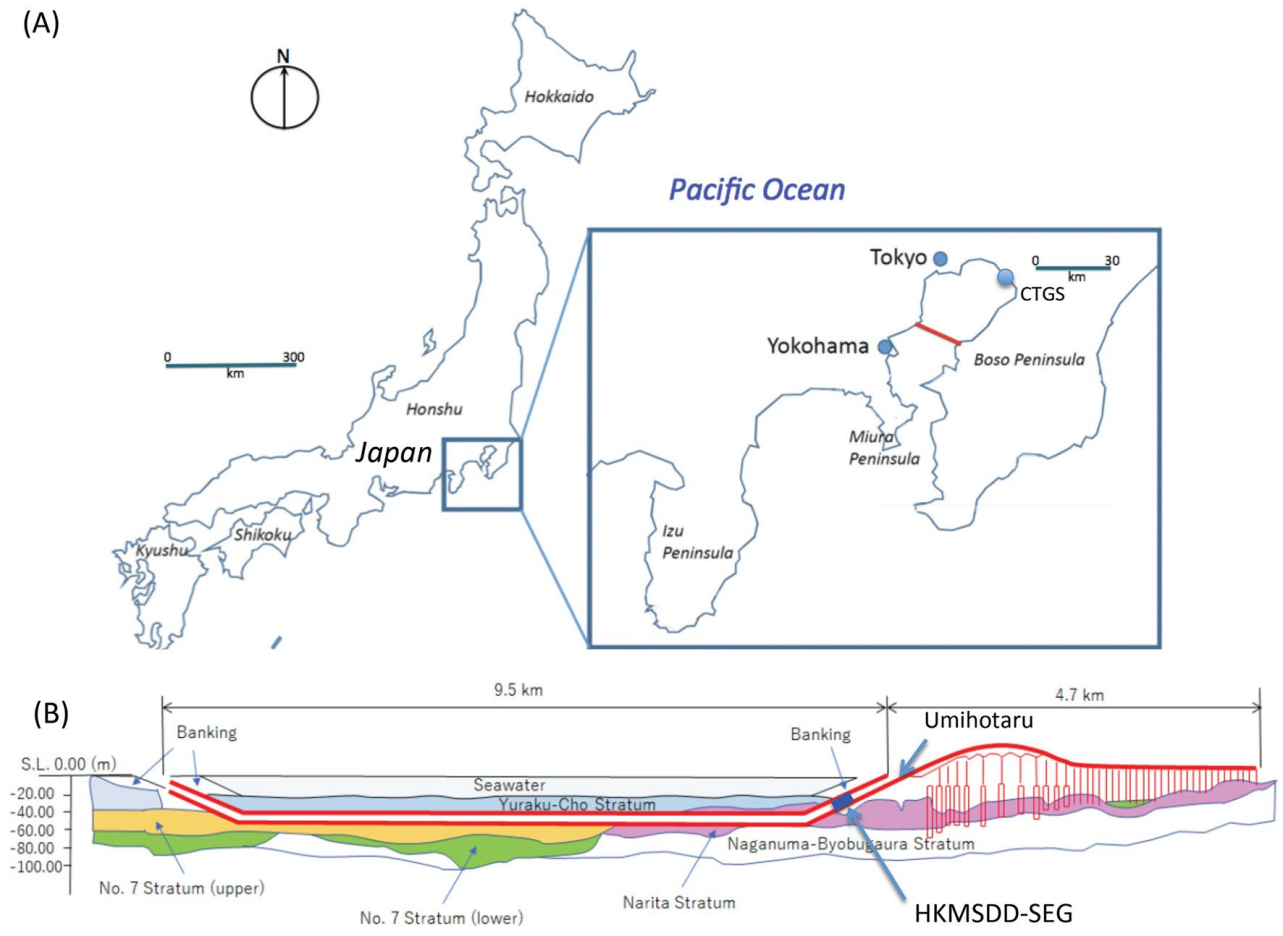
Location of the Tokyo Bay Aqua-Line (TBAL) in Japan (red lines) (**A**) and the cross-sectional view of the tunnel section (Aqua-tunnel) of TBAL (**B**). The symbols CTGS and HKMSDD-SEG respectively indicate the locations of the Chiba tide gauge station and the HKMSDD segment. The name Umihotaru indicates the service area that marks the transition between the bridge and tunnel part. HKMT drew the map and the image with Microsoft PowerPoint software and holds the copyright.

**Figure 3:**
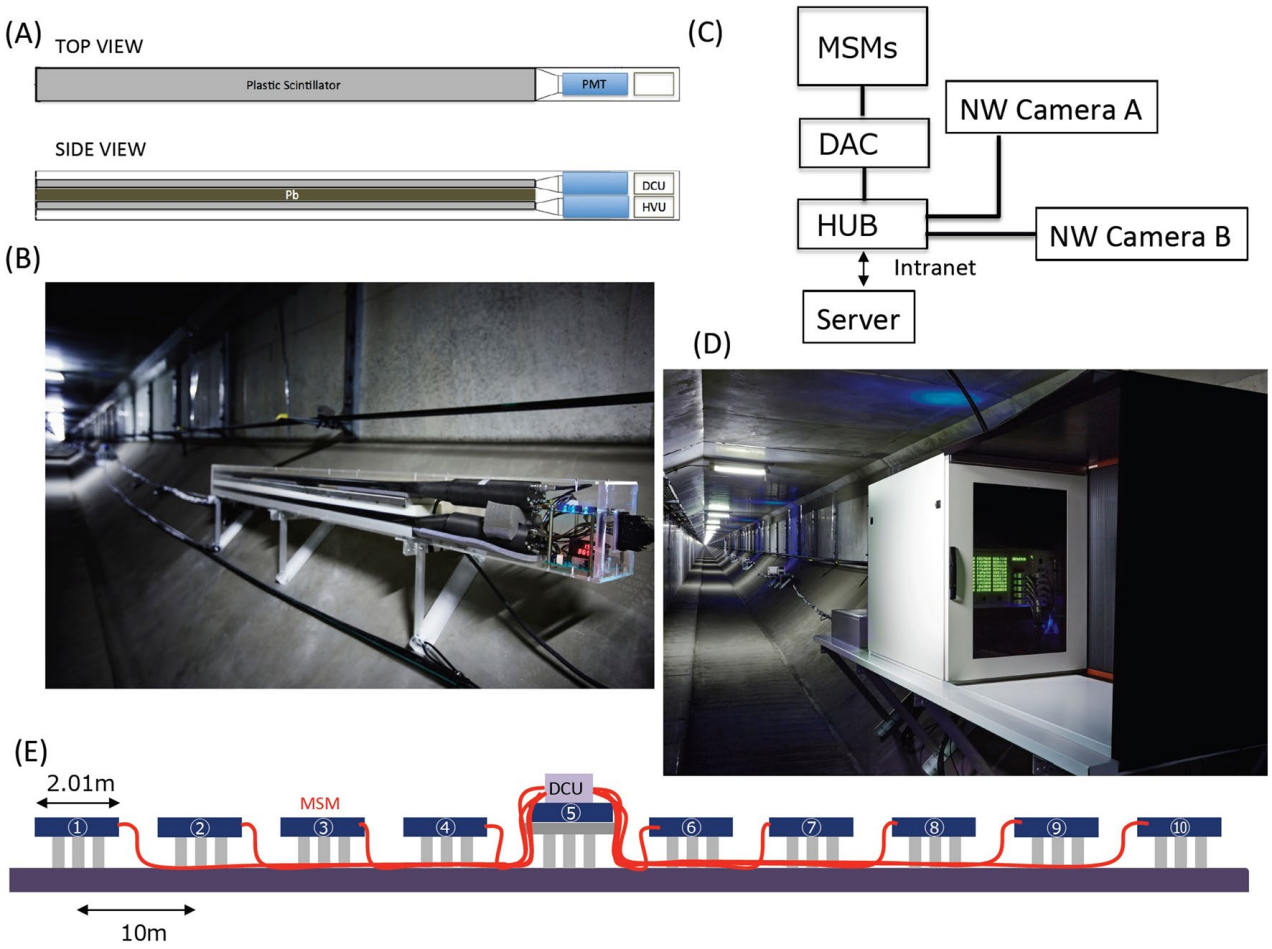
Configuration of the HKMSDD segment. The schematic of the muographic sensor module (MSM) (**A**) is shown along with a photograph of the MSM (**B**). The abbreviations DCU and HVU respectively indicate the discriminator-coincidence unit and the high-voltage power supply unit. The block diagram of the data collection scheme (**C**) is shown along with a photograph of the Data Acquisition Center (DAC) (**D**). The network cameras (NW Cameras) are respectively used for monitoring the MSM LEDs and the DAC 7-segment LEDs. The HKMSDD server hosts a closed network. The schematic view of the HKMSDD segment is also shown (**E**). HKMT holds the copyright of the photographs.

The Tokyo Bay Aqua-Line (TBAL), which is also known as the Trans-Tokyo Bay Expressway, is a combined bridge and tunnel structure spanning the entire width of Tokyo Bay, Japan (Fig. 2). Tokyo Bay has some of the highest maritime traffic sea channels in the world, and 500–1000 ships travel over or under the span of TBAL every day. TBAL consists of a 4.4-km long bridge (30% of TBAL) and a 9.6-km long tunnel (70% of TBAL) underneath the bay. The tunnel section is called the Aqua-Tunnel. The average sea depth is 20 m in most of the region where the Aqua-Tunnel was constructed, and the tunnel was constructed at a depth of 20 m underneath the seafloor. In this work, 30 muographic sensor modules (MSMs) were deployed inside the Aqua-Tunnel to construct a linear array of MSMs called the Tokyo-bay Seafloor Hyper-Kilometric Submarine Deep Detector (TS-HKMSDD). Each MSM consists of two scintillation detectors, a high-voltage power supply unit (HVU) (Technoland Z-SYS 070HV), and a discriminator-coincidence unit (DCU) (Technoland Z-SYS 070DC) (Fig. 3). When a muon passes through the detector, the scintillation light is transported to the photomultiplier tube (PMTs) (HAMAMATSU R7724 ASSY) attached to these plastic scintillators (10 cm wide, 150 cm long) (ELGEN EJ-200) via acryl light guides (Fig. 3A). The signal outputs from these PMTs are transferred to the DCU to select only the events that pass through both of these scintillators at the same time. Although we did not expect secondary cosmic charged particles at a depth of more than 40 m water equivalent (m.w.e.), a 2-cm thick lead block was inserted between these plastic scintillators to remove background radiation that could emit from the concrete wall of the tunnel (see “Method” section for a more detailed description about the MSM design). Tokyo Bay was chosen for conducting submarine muography because various maritime data sets including seawater and atmospheric temperature data, barometric data, salinity data, wind speed and direction data, precipitation data, humidity data, etc. are available, enabling more comprehensive comparison and evaluation of our muographic results.

An HKMSDD segment consists of ten MSMs, each spaced evenly at an interval of 10 m and the data acquisition center (DAC) located at the center of the segment (Fig. 3E). Since the size of the scintillator of each MSM is 1500 cm^2^, each HKMSDD segment has an active area of 1.5 m^2^. Each segment measures 100 m in length (see “Method” section for more detailed description about the HKMSDD segment). In this work, one HKMSDD segment (SEG) was installed in the Aqua-Tunnel. The SEG was placed within the region 500–600 m from the station called Umihotaru (the Japanese word for Vargula hilgendorfii, a local bioluminescent, ostracod crustation) that marks the transition between the bridge and tunnel part. Umihotaru is located in the middle of Tokyo Bay, and is located at almost the same distance from three major coastal cities (30 km from Tokyo city, 27 km from Chiba city and 22 km from Yokohama city) (Fig. 2A). The nearest neighbor tide station (CTGS) was located at the Chiba port (25 km NE of Umihotaru). Directly above the tunnel in which the SEG is installed is a 10 m thick rock overburden (the seabed) and approximately 20 m of water (Fig. 2B). The operation of the SEG started on March 5, 2021. The ATH data were collected after June 1, 2021. The muon signals detected by MSMs are transferred to DAC, counted, and transferred in real time to the external server located in Kyoto, Japan. These numbers of counts are also locally displayed on the LED panel attached to DAC. Each DCU has an LED light that flashes when the detector is triggered, and both local LED panels and LED lights are monitored with the surveillance camera for remote monitoring.

**Figure 4:**
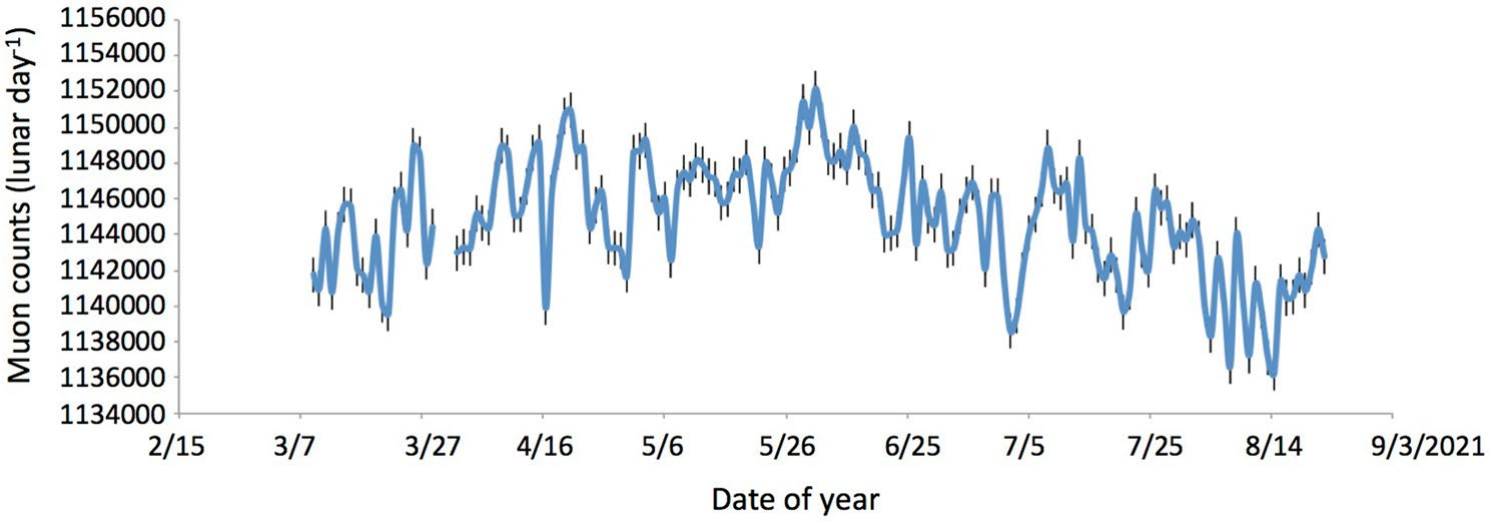
Time-sequential plot of the lunar-daily muon counts. The error bars indicate the statistic errors (1σ) associated with the data points.

Figure 4 shows the time-sequential plot of the lunar-daily (24 h and 50 min) muon counts. Here, the lunar day refers to the period between moonrises. Therefore, by taking the lunar-daily average, instead of a standard day of 24 h, variations in the astronomical tide levels are canceled. The muon rate is almost constant and the average muon counts per lunar day and standard deviation (S.D.) were respectively 1,144,288 and 3187 (~ 2.8 per mille).

**Figure 5:**
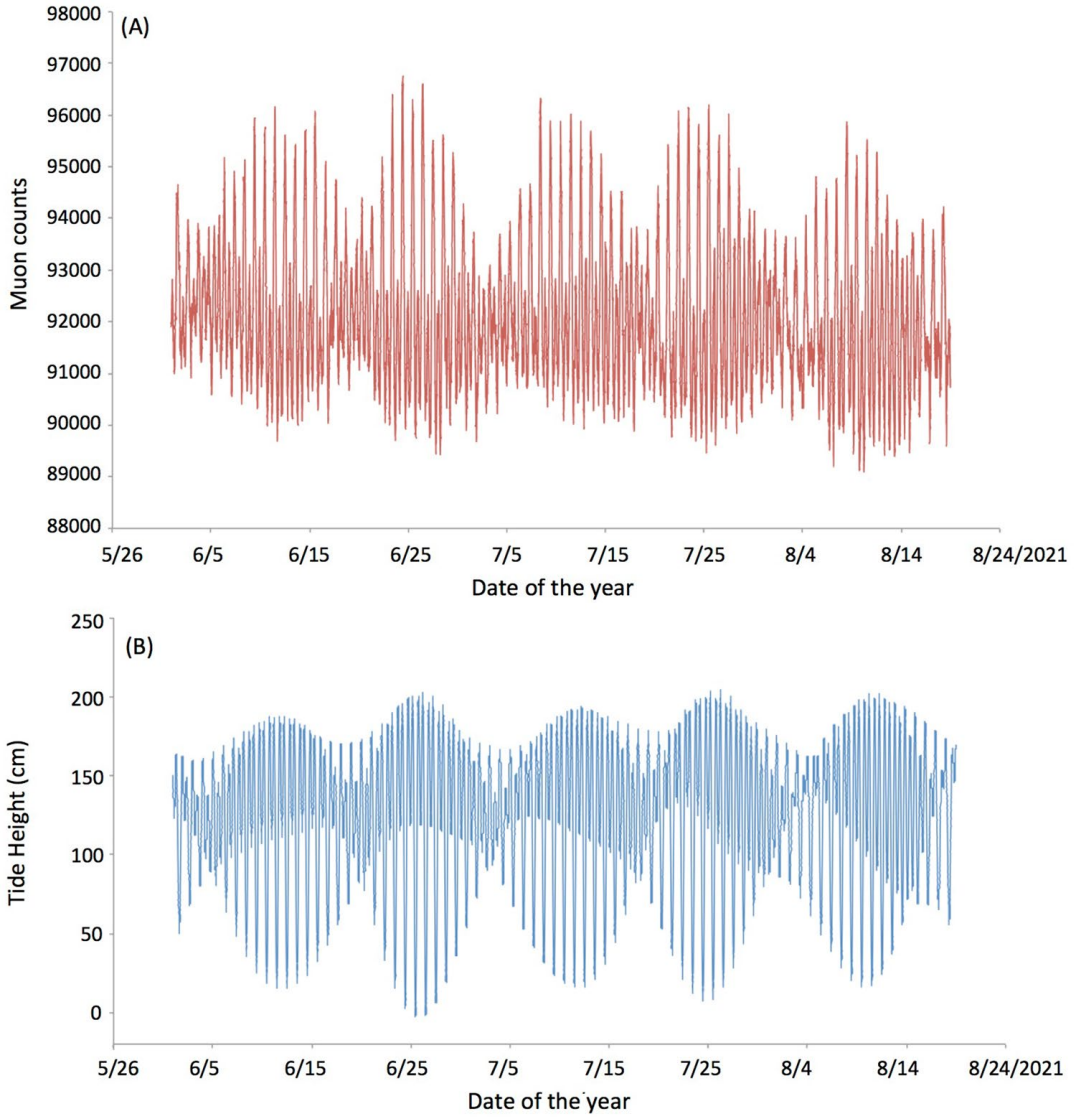
Time-sequential plot of the number of muon counts collected every 5 min (**A**) and the ATH variations (**B**)^36^.

Variations in the atmospheric pressure are compensated with the column density of seawater. The inverse barometer effect (IBE) compensation is approximately correct because the energy loss per unit mass differs between air and water. For example, while the CSDA range is 1.845 × 10^4^ g/cm^2^ for 4 GeV muons in air, it is 1.810 × 10^4^ g/cm^2^ for the muons in water with the same energy^35^. However, this 2% difference is negligible in the current work. Variations in the atmospheric pressure are up to 50 hPa that is equivalent to a water thickness of 50 cm. Since this 2% difference is applied to this water thickness, the uncertainty in sea level gauging that comes from this difference is much smaller than the resolution of our current system. The seawater density depends on salinity, temperature and pressure (S, T, P). However, in general, S–T–P-driven water expansion is not isotropic, and in particular, it is difficult to predict the local variations in the sea level. This effect will be further discussed in our future work with data as obtained from a longer observation period (> 1 year). Figure 5A shows the time-sequential plot of the number of muon counts collected every 5 min. The data points were smoothed by taking a moving average with a time window of 2 h. The daily tide variations along with a longer cycle of spring and neap tides are clearly seen as an anti-correlation with ATH (Fig. 5B). When the tide level rises, the muon counts decrease and vice versa. The height difference between high and low tides during the neap tide period was 20–30 cm, and this level of tide variations can be clearly distinguished with the current HKMSDD segment.

**Figure 6:**
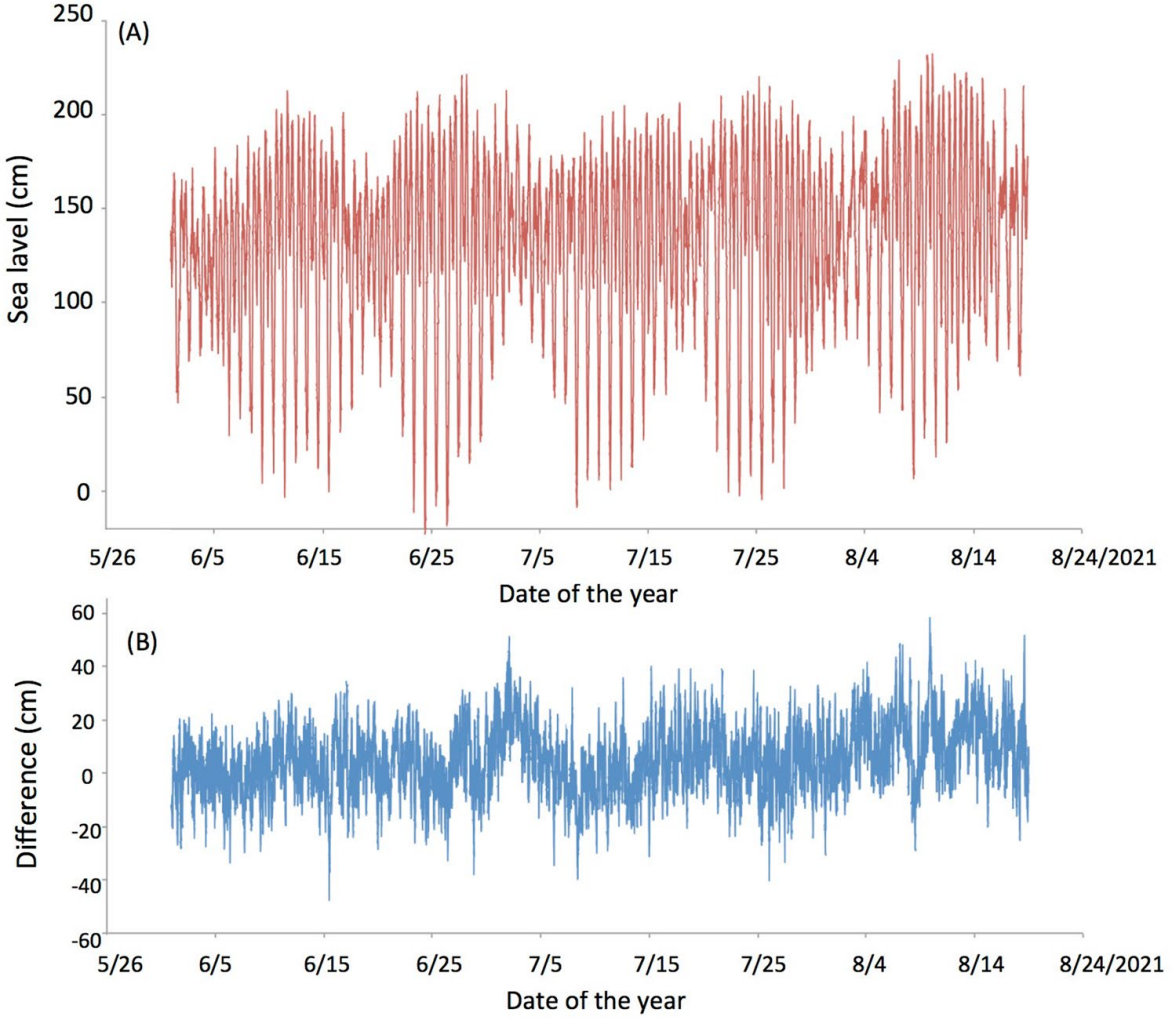
Sea levels converted from the muon counts (**A**) and their difference from the ATH variations (**B**).

**Figure 7:**
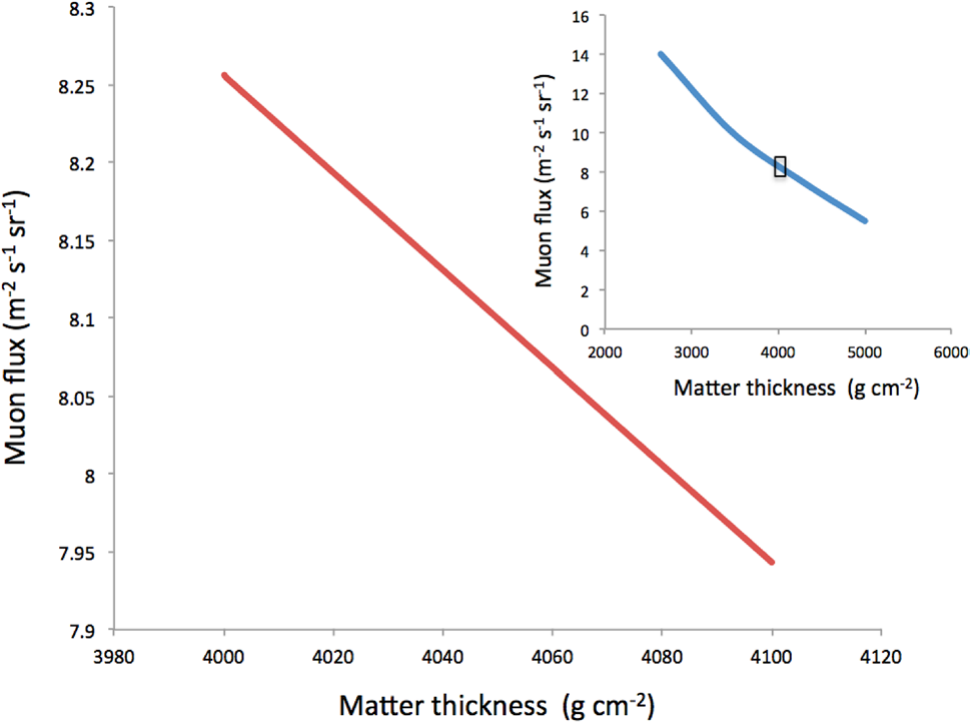
Muon flux expected at the Hyper-Kilometric Submarine Deep Detector (HKMSDD) segment placed within the region 500–600 m from Umihotaru (SEG1) for various matter thicknesses. The inset shows the larger scale plot. The rectangular box in the inset indicates the plotting region of the main graph.

Figure 6 compares the sea levels converted from the muon counts with ATH (Fig. 5B) during the period between June 1, 2021 and August 18, 2021. Since muography measures the integrated density along the muon paths, the local seafloor topography and the average density of the seabed above the tunnel affects the absolute sea level gauging. For this reason, the technique to measure the relative tide variations without the necessity of the knowledge of these external factors was developed by using a simple relationship between muon flux and matter thickness they traverse (see Fig. 7).

In muography, the muon range as a function of the incident muon energy is incorporated in the open-sky muon spectrum as a function of the zenith angle (*θ*). Once both the muon path length and the average density along the path are known, the thickness (*X*) can be calculated by multiplying them, and thus the minimum energy (*E*_c_) of muons that can penetrate through a material. By integrating the open-sky spectrum *I* (*E*, *θ*) from *E*_c_ to infinity, we obtain the integrated muon intensity *N*(*E*_c_,), which represents the number of muons that have enough energy to escape from the target of interest:

1 $$N(E_{c} ,\theta ) = \int_{Ec}^{\infty } {I(E,\theta )} dE,$$

where *I*(*E*, *θ*) is the zenith-angle-dependent open-sky muon energy spectrum, and *E*_c_ is the cutoff energy as a function of thickness, *X*. More detailed descriptions about this principle in muography can be found elsewhere^14^. The continuous slowing down approximation (CSDA)^35^ can be applied in the present case due to the shallow depth. Due to the geometric acceptance of the detector, and the slant depth of both seawater and seabed, most muons arrive from the near vertical direction (50%, 70%, and 90% of the total muons respectively arrive within the angle region 0°–20°, 0°–33°, 0°–50° from zenith). The integrated muon flux calculated with Eq. (1) is shown in Fig. 7 for small variations in the matter thickness which the muons traverse. The vertical spectrum^37^ was used, and the lower cutoff energy for the given water thickness were based on the CSDA muon range^35^. In this plot, the range is focused on values between 4000 and 4100 hg cm^−2^. As shown in this figure, if the deviation (∆*X*) in *X* in Eq. (1) is significantly smaller than *X*, the relative penetrating muon flux (∆*N*) which can be expressed as:

2 $$\Delta N = k\Delta X + C$$

is a linear function of ∆*X*. By utilizing this flux-thickness relationship, the muon counts were calibrated with the ATH variations as reference data and only the relative difference (∆*X*) was considered for conversion from the muon counts to the tide height. This method has an advantage for cases when we do not have accurate information about the factors that increase the uncertainty in the total rock-water thickness *X*. The HKMSDD data on June 1 were fitted to the ATH data to adjust the coefficient, *k*, and the constant, *C*, in Eq. (2) to reproduce the tide levels of the rest of 79 days. The average and standard deviation of the difference between the reproduced 22,752 data points and ATH was 12.85 cm.

## Discussion

It was shown that muography has the potential to provide the variations in the thickness of the water column located above the detector. This technique enables us to measure tide levels at the locations where the conventional tide measurements are difficult to conduct. However, since muography measures the integrated mass above the detector, the measurements are influenced by some factors that do not have to be considered with the conventional techniques.

In the near future, TS-HKMSDD has been planned to extend to the full scale of 9.6 km throughout the Aqua-tunnel. In this section, we discuss how the undersea environment can possibly affect the muographic measurements and how these errors can be corrected or suppressed. After that, we summarize our prospects based on the potential of the proposed full-scale detector array.

### Atmospheric effect

**Figure 8:**
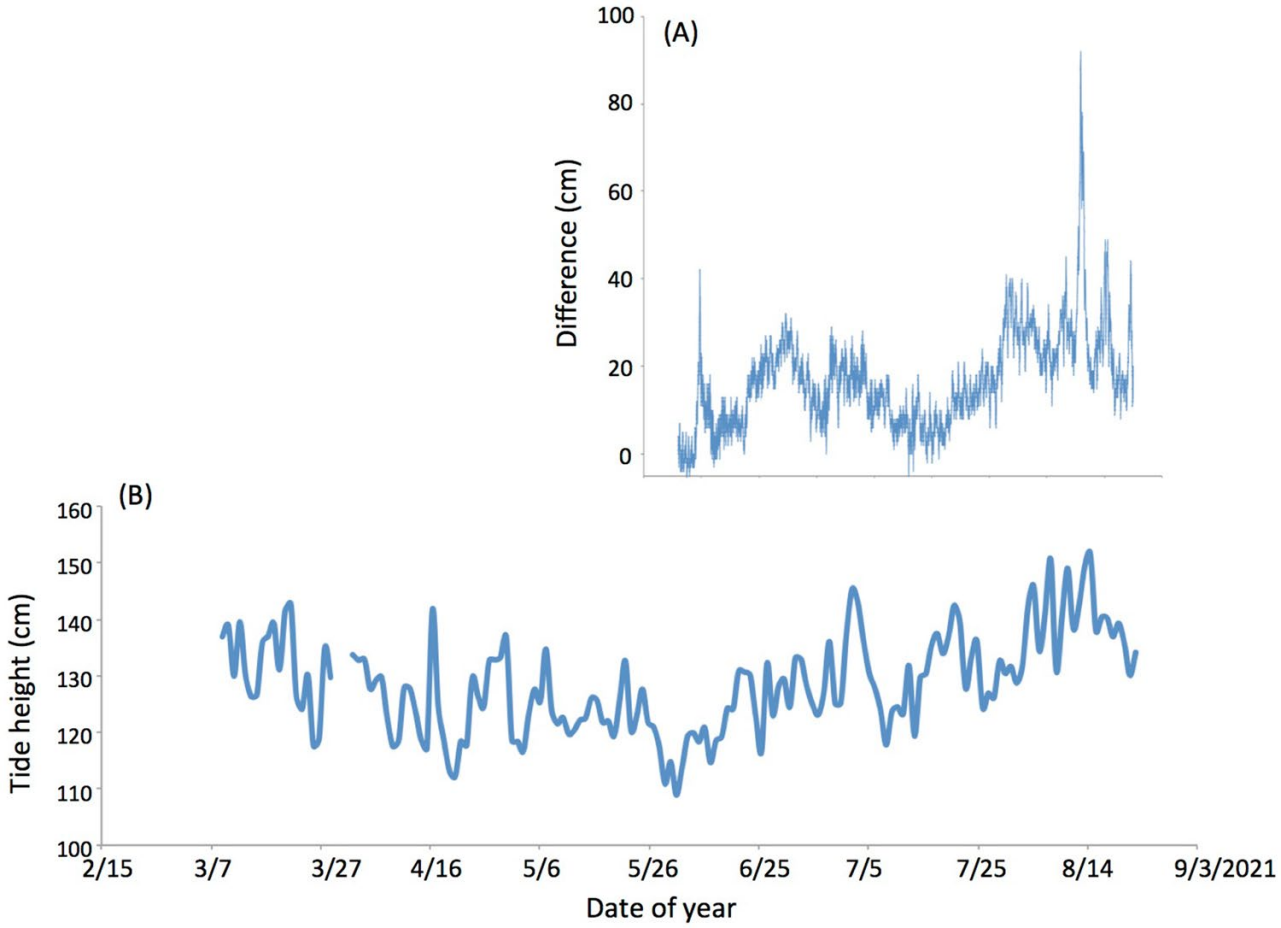
Difference between the Chiba tide gage station (CTGS) data and ATH (**A**). The lunar daily averaged muographic tide height variations (**B**) are plotted together. The time axes are common between these two plots.

In the case of submarine muography, the muon flux is not strongly affected by the atmospheric pressure variations since fluctuations in the sea level have a complementary relationship with the atmospheric pressure fluctuations above. This effect is called inverse barometer effect (IBE). If the atmospheric pressure is reduced by 1 hPa, the sea level rises by 1 cm. On the other hand, for the purpose of the muographic tide gauging, there is a contributing factor to the actual tide height. In Fig. 8A, the difference between the CTGS data and ATH data (CTGS-ATH) is shown. In Fig. 8B, the time-sequential plot for lunar daily averaged muographic sea levels is shown. The same parameter to plot Fig. 6 was applied. The energy of muons must be at least 10 GeV at sea level to reach the detector. The energy loss difference varies sharply in the 1–10 GeV range due to the density effect. Considering a maximum difference of 8% together with a 50 cm IBE, this could lead to a 4 cm correction to the IBE height. The positive correlation between the muographic data and IBE in Fig. 8 might have reflected this effect. This effect will be further studied with future additional detectors. There is also a stratospheric temperature effect on the muon flux^38–40^. This effect generally affects high-energy muons with energies above tens of GeV^41^, and may affect the seasonal flux of muons with energies (~ 10 GeV) we are discussing here. This effect will be further studied with longer period measurements.

### TS-HKMSDD as a tide monitor on the heavy traffic waterway

Undersea tunnels are constructed to allow shipping to pass above them without traffic congestion. Bridges with opening or swinging mechanisms also allow shipping to pass, but they can cause traffic congestion. Moreover, longer bridges are more difficult to open or swing. Alternatively, building higher bridges will help to avoid having to add opening and swinging mechanisms; however, these bridges are more costly and more fragile against high winds. Inversely, the locations where the undersea tunnels were constructed are prone to having both heavy automobile and maritime traffic. Therefore, it is difficult to perform conventional tide measurements with buoys in the waters above these undersea tunnels in spite of the advantages they provide for the safety of the navigation. As a consequence, there is a clear incentive to investigate if muography can be used to fill in this gap and conduct tide measurements in heavy traffic waterlines that are now vulnerable.

Urbanizations have historically occurred in bay areas because they are relatively safer against storm surges in comparison to land facing the open ocean. Storms occurring over the open ocean can generate large waves called swells. Large swells generated by the storms propagate outward from the storm, traveling over long distances across the ocean, but their energy is conserved or weakly dissipated during their propagation, and they release most of their energy on the open coasts with high waves (sometimes more than 10 m). The negative effects of large swells are generally mitigated by using some sort of blocking mechanism, like narrow entrances that characterize many bays^42^ throughout the world. However, some of the swells can nonetheless pass through bay entrances, and may consequently cause damage to the coastal infrastructure. For example, the largest presence of such bay swells occurs in the region of Central Bay between the Bay Bridge and the Richmond-San Rafael Bridge, San Francisco, CA^43^. Another region that sometimes attracts extraordinarily large waves is Tokyo Bay. For example, the swells generated by Typhoon-15 reached all the way to Tokyo Bay and damaged the breakwater of Yokohama port in 2019. After analyzing the meteorological data collected there, it was found that the ocean-driven swells had overlapped with the local waves and the wave height reached 5.57 m. By considering the astronomical tide and the wave pressure applied to the breakwater, it was estimated that the maximum height of the waves reached 9.18 m at the coast^44^. As is evident in such an example, these ocean-driven swells have a potential impact on coastal land. However, the swell heights are difficult to predict^45^. Utilizing trans-bay undersea tunnels, muography offers a solution to measure the thickness of the water column located above the tunnel on heavy traffic water lines from locations underneath the seafloor. This technique is applicable to waterways throughout the world with undersea tunnels.

**Figure 9:**
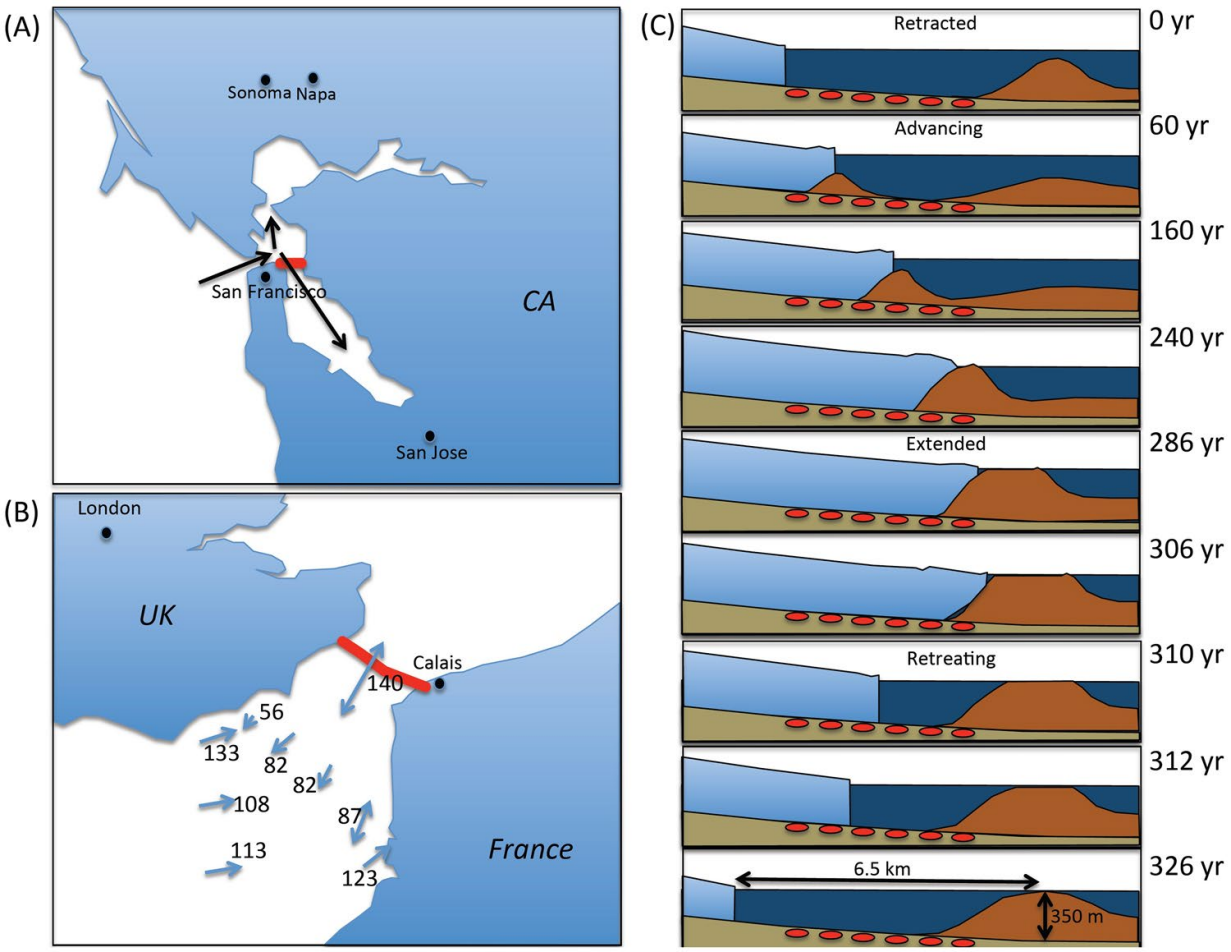
Possible future HKMSDD deployment sites: (**A**) Transbay Tube, San Francisco, CA, (**B**) Channel Tunnel, UK/France, and (**C**) Glacier Upsala, Patagonia, Chile. Red lines in (**A**,**B**) indicate the undersea tunnels. Black arrows in (**A**) indicate the direction of the pacific swells^43^. Blue lines in (**B**) indicate the spring maximum current vectors. The numbers indicate the speed in units of cm/s^60^. Red oval shape symbols in (**C**) indicate the hypothetical HKMSDD buried under the seafloor. HKMT drew the maps in (**A**,**B**) with the Microsoft PowerPoint software and holds the copyright. HKMT drew the image in (**C**) based on the work by Brinkerhoff et al.^68^.

Understanding of the regional tide streams in the inner bay is important for the safety of navigation and environmental assessments, as well as studying about the regional seawater circulation types and pollution distribution. For this reason, tidal flow fields have been numerically modeled in various waters^46–50^. In order to conduct the tidal stream simulations, the boundary conditions have to be set between the region in which the simulations are conducted and the coast or the region in which the simulations are not conducted. In these simulations, which are designed to reproduce the tidal stream as a result of tidal level variations, tidal levels are given as the boundary condition, and therefore the accuracy of these values will directly influence the quality of the simulations. Conventionally, the tidal data obtained from the tide gauge stations have been used as the boundary condition. However, since the locations of these tide gauge stations are spatially dispersed, the data have to be interpolated. As a result, the interpolated data have a tendency to be more erroneous as a function of the distance from the stations. In order to solve this problem, other researchers have developed numerical-model-based interpolation methods^51^. Undersea muography will provide complementary boundary conditions to apply to these tidal flow field simulations. A full-scale extension of TS-HKMSDD will constrain the modeling results of the tidal stream in Tokyo Bay. The final deployment of ~ 100 HKMSDD segments across Tokyo Bay would directly provide the open boundary condition for the modeling of the northern half of Tokyo Bay. The HKMSDD segments are applicable to other undersea tunnels in the world. For example, the Transbay Tube in San Francisco, CA (Fig. 9A).

### TS-HKMSDD as a tsunami monitor

Submarine earthquakes sometimes cause large seafloor displacements, which subsequently may generate tsunami events. A minimum triggering earthquake magnitude of 5.5 is suggested for a sufficiently large submarine event which could generate a devastating tsunami if the epicenter is located within the continental slope^52^, otherwise a larger magnitude of 7.0–7.5 is required^53,54^. A tsunami is a large ocean wave that can be triggered by earthquakes, landslides, and mountain collapses. Tsunami waves generally have average wavelengths of 500 km with an initial propagation speed of more than 200 m/s^55^, but since the speed tends to decrease as these waves approach coastal zones, the successive waves overlap, causing wave height to increase. Thus, the tsunami is potentially catastrophic to coastal cities. Another contribution of TS-HKMSDD is to monitor tsunami propagation. Detecting the tsunami and imaging of the wave height distribution prior to its arrival to the seacoast may be possible. It has been predicted that a future large earthquake called the Nankai Trough Earthquake with an assumed magnitude of 9.1^56^ may cause tsunami waves to arrive at the coast of Tokyo Bay with a height of 2.48 m in Koto, 2.46 m in Chuo, 2.44 m in Shinagawa, 2.40 m in Minato, 2.37 m in Ota, 2.07 m in Edogawa and 1.88 m in the reclaimed land of Tokyo bay (such as Odaiba and Palette Town), respectively^57^. It may be possible to detect this tsunami 30–40 min prior to arrival at the Tokyo coastal area assuming that the tsunami travels at 30 km/h. The actual height distribution and speed of a tsunami above the Aqua Line tunnel will provide quantitative information of the impending tsunami before it reaches the inner parts of the bay area.

### TS-HKMSDD as a sediment accumulation monitor

In the coastal area of Tokyo Bay, many estuaries have been urbanized with heavy traffic in the waterways. There are dozens of ports including three major ports in Tokyo Bay: Tokyo, Yokohama, and Yokosuka ports. In these ports, the navigation channels and harbor basins are getting shallower due to the deposition of discharged sediments from the rivers. These sediments need to be continuously removed from these channels. As a consequence, periodic dredging is required to maintain these navigation channels and harbors in a safe and usable way. Dredging is the excavation of materials from a sea, river, or lakebed and depositing them at a new location for the purpose of reshaping land and water features to alter drainage, navigability, constructing dams, flood and storm protection, and other controls for streams and shorelines^58^. However, dredging activities have potential environmental impacts, either within the dredging area or the deposition site. The dredging process causes sediment resuspension that increases the turbidity of water as well as nutrients and pollutants dispersion, the latter that can cause local water contamination, and possible ecosystem interference (by burying biological habitats, for example). This problem is not restricted to Tokyo Bay, but it is a global issue, applicable to many of the world’s ports. Determining the optimized timing and the amount of dredging is essential to minimize the impact on the environment. However, the sedimentation rate varies depending on many factors including rainfall, seasonality, geology, topography, sea current, etc. and thus, it is difficult to numerically estimate it. Currently, there is no in-situ method to monitor the rate of this deposition.

Sediment monitoring also provides useful insights into the functioning and health of an estuary. Recent studies have shown that sediment loads delivered to estuaries have rapidly increased as a response to the erosion caused by the loss of native vegetation when farms^59^ or urban areas^60^ are introduced to a landscape. Although this estuary-filling rate is particularly accelerated following intense rainfall events, it has been found that in some estuaries this rate has further accelerated during the last few decades in comparison to earlier in the last century^61^. Compared to the vertical accumulation rate, the sediment mass accumulation rate provides more quantitative information about the contamination. There are density variations depending on the depth of the estuarine sediment. These variations are reflected by compaction or changes in the composition of the sediment^62^. Muography will offer a novel method to monitor the sedimentation rate on the seafloor if the mass-driven sea level variation rate (due to the influx from land) is sufficiently smaller than the sedimentation rate.

A fast sedimentation rate is not only occurring in regions near estuaries. A fast tide current also accelerates the sedimentation. For example, in the Kanmon channel, Japan, the seafloor was dredged twice in a last couple of decades (1.5 m in 2001 and 2.0 m in 2006)^63^. The Kanmon channel is used for short-cutting the commercial waterlines between Japan and the Eurasian continent. Consequently, extensive traffic control is necessary to process boats that pass through this channel every minute. In 1958, a tunnel was constructed under this channel (Kanmon tunnel) to mitigate the land traffic between Honshu Island and Kyushu Island, Japan. In the near future, Kanmon Tunnel HKMSDD will be installed to monitor the sedimentation on the seafloor.

It is also known that fast tidal current of more than 200 cm s^−1^ has been observed in the English Channel. As a result, giant tidal dunes are generated and transported on the seafloor. Most of these sand bodies have been studied with side-scan imagery, high-resolution seismic surveys, and bathymetry^64,65^. All these studies demonstrate the high mobility of these sand bodies. For example, in the western channel, 8 m high asymmetric dunes have been monitored for 4 years and showed an average migration of 20 m year^−1^ in the direction of the dominant tidal current to the north. As can be seen in Fig. 9B, one of the fastest spring current speed was observed above the Channel Tunnel. HKMSDD installed in the Channel Tunnel can be used for monitoring such a large-scale migration of sand bodies in real time to understand present-day seafloor dynamics.

Imaging tidewater glaciers (TWG) with muography would contribute to our understanding of glacier cycles in general and their effect on recent climatic global issues. It has been documented that most glaciers are melting and shrinking^66^, although some tidewater glaciers (TWG) have actually been expanding in size recently^67^. In order to understand this trend, it is important to know the processes and time scales of sediment production, transport, and accumulation near the ablation fronts of retreating tidewater glaciers. In particular, sedimentation transported to the sea via tides affects the tidal water glacier periodicity. For example, Taku glacier, Alaska, has advanced by 5 km since 1933^68^ while Glacier Upsala, Patagonia, is experiencing rapid calving retreat^69^. Brinkerhoff et al.^68^ modeled the periodic cycles of TWG glaciers in the following way. Sediment carried to the terminus is transported to the sea and deposited with a rate of ~ 1 m year^−1^, forming a shoal. The shoal acts as a plug that prompts thickening and promotes the growth of the glacier onto the shoal, but eventually a void at the upstream end of the shoal opens due to its basal motion, and the ice and bed decouple, driving glacier retreat that continues over decades until it is ready to begin the cycle anew. This process repeats every ~ 300 years. Although it is unlikely that there are tunnels already constructed underneath the seafloor near the downstream end of a glacier, if we position HKMSDD there with optical submarine cable at the downstream end of the retreating glacier (Fig. 9C), e.g., Glacier Upsala, real-time monitoring of the aforementioned process will be possible.

In conclusion, we successfully obtained and reported the first result of undersea muography with TS-HKMSDD. The detector has been operating in stable condition since March 5, 2021, and the lunar-daily-averaged muon rate showed small fluctuations with 2.8 per mille S.D. The 5-min muon rate showed clear anti-correlation with ATH with a clear differentiation of the large tide from the small tide (20–30 cm) in the period of neap tide. The results indicated that in the future, utilizing an undersea tunnel for muography may offer a practical solution for tide measurements in important locations such as near heavy maritime traffic waterlines where the conventional tide monitoring is difficult. Moreover, undersea muography will offer a solution to monitor the sedimentation process on the shallow seafloor where acoustic methods are difficult to implement. It is currently planned that UK-HKMSDD will be deployed in an undersea tunnel in the UK to compare the results under the conditions of a different undersea environment. We anticipate that further knowledge and experience in undersea muography will be accumulated as other HKMSDDs are installed in tunnels worldwide for the application of this technique to imaging targets such as tides, natural resources and the seafloor topography of coastal regions.

## Method

### Muographic sensor module

The muographic sensor module (MSM) consists of two scintillation detectors, a high voltage power supply unit (HVU), and a discriminator-coincidence unit (DCU). Each scintillation detector consists of a plastic scintillator (ELJEN EJ-200) with dimensions of 20 mm in thickness, 100 mm in width and 1500 in length that is coupled with a photomultiplier tube (PMT) (HAMAMATSU H7195) via an acryl light guide. The HVU (Technoland Z-SYS 070HV) is a 2-channel high voltage power supply that can apply a high voltage ranging 0–2000 V to 2 PMTs of the MSM. A 7-segment red LED panel attached to the HVU displays the value of the voltage applied to the PMTs (Fig. 3B). The DCU (Technoland Z-SYS 070DC) consists of a 2-channel discriminator and a coincidence circuit. When the DCU triggers the signal, the attached blue LED lights up (Fig. 3B). The input signals are negative analogue pulses and the output signals are RS422. The input connecter type is LEMO and the output connector type is D-SUB 25 PIN. The MSMs are anchored to the tunnel wall with bolts and frames to fix their position.

### HKMSDD segment

An HKMSDD segment consists of 10 MSMs with an interval of 10 m and the data acquisition center (DAC) located at the center of the segment. Each MSM is connected to DAC with the water-resist D-SUB cables (IP67). The cable lengths are different, depending on the distance of MSMs from DAC, and a pair of 10-m, 20-m, 30-m, and 40-m cables are used. Due to the asymmetric positions of DCU within the HKMSDD segment, the lengths of the cables to the first and 10th detectors are different: 50 m and 55 m. DAC consists of (A) four complex programmable logic devices (CPLD) (Intel 10M08), (B) three green LED panels (Rohm LAP-301 ML), (C) a 10-MHz temperature compensating clock (TCC) (Mita-Dempa MX033-0510-10 MHz), (D) switched mode power supply (SMPS) (Technoland Z-SYS 070PS), and (E) a microcomputer board (Raspberry Pi 4). (A) is used for counting the number of signals output from DCU. (B) is used for displaying the number of counts. Each LED panel has 10 8-digit, 7-segment displays. Three LED panels respectively count the number of single and coincidence counts. (C) is used for measuring the time. A heating device is attached to the clock and the temperature-dependent variation is suppressed to less than 10 ppm for the seasonal temperature variation. (D) is used as a power supply for HVU and DCU. (E) is used for sending the data to the external server. An electric cable and an 8-core optical fiber cable were used for connect between DAC and the electric room of the Aqua-Line to supply electric power to the HKMSDD segment and to transfer the collected data including the text and video data to the external server. The network speed was 1 Gbps on a best-effort basis.

## Supplementary Information

Supplementary Information.
