# Author Correction: First results of undersea muography with the Tokyo-Bay Seafloor Hyper-Kilometric Submarine Deep Detector

**DOI:** 10.1038/s41598-021-01979-9

**Published:** 2021-11-11

**Authors:** Hiroyuki K. M. Tanaka, Masaatsu Aichi, Cristiano Bozza, Rosa Coniglione, Jon Gluyas, Naoto Hayashi, Marko Holma, Osamu Kamoshida, Yasuhiro Kato, Tadahiro Kin, Pasi Kuusiniemi, Giovanni Leone, Domenico Lo Presti, Jun Matsushima, Hideaki Miyamoto, Hirohisa Mori, Yukihiro Nomura, László Oláh, Sara Steigerwald, Kenji Shimazoe, Kenji Sumiya, Hiroyuki Takahashi, Lee F. Thompson, Yusuke Yokota, Sean Paling, Dezső Varga

**Affiliations:** 1 University of Tokyo, Tokyo, Japan; 2 The University of Salerno, Salerno, Italy; 3 Istituto Nazionale di Fisica Nucleare-Laboratori Nazionali del Sud, Catania, Italy; 4 Durham University, Durham, UK; 5 Kerttu Saalasti Institute, University of Oulu, Oulu, Finland; 6 Muon Solutions Oy Ltd, Saarenkylä, Finland; 7 Arctic Planetary Science Institute, Rovaniemi, Finland; 8 NEC Corporation, Tokyo, Japan; 9 Kyushu University, Fukuoka, Japan; 10 The University of Atacama, Copiapó, Chile; 11 The University of Catania, Catania, Italy; 12 Istituto Nazionale di Fisica Nucleare, Catania, Italy; 13 Kansai University, Osaka, Japan; 14 The University of Sheffield, Sheffield, UK; 15 Geoptic Ltd, London, UK; 16 Boulby Underground Laboratory, Loftus, UK; 17 Wigner Research Centre for Physics, Budapest, Hungary; 18 International Virtual Muography Institute (VMI), Global, Tokyo, Japan

Correction to: *Scientific Reports* 10.1038/s41598-021-98559-8, published online 30 September 2021

In the original version of this Article, Masaki Satoh was incorrectly listed as an author of the original Article, and has subsequently been removed.

The original Article has been corrected.

